# The Effects of Ultra-Processed Food Consumption—Is There Any Action Needed?

**DOI:** 10.3390/nu12092556

**Published:** 2020-08-24

**Authors:** Anna Gramza-Michałowska

**Affiliations:** Department of Gastronomy Sciences and Functional Foods, Faculty of Food Science and Nutrition, Poznań University of Life Sciences, Wojska Polskiego 31, 60–624 Poznań, Poland; anna.gramza@up.poznan.pl

In recent years, there has been an increased interest observed concerning the relationship between the consumption of highly processed foods and health impact. The growing incidence of chronic diseases in modern society has become a relevant and current topic. Therefore, it is necessary to consider how to reverse the trend of the increased consumption of ready-to-eat and convenient food and return to the well-established eating patterns based on minimally processed foods and freshly prepared meals.

The results of numerous studies support the claim that the orientation of global food supply towards ultra-processed foods may partly explain the growing trends in the incidence of chronic non-communicable diseases and an overall higher mortality risk [1,2,3,4]. Therefore, the perspectives on the soaring popularity of highly processed foods relate mainly to health and technological aspects. A properly balanced diet is an important element in the prevention of civilization-related diseases such as cardiovascular disease, diabetes and obesity. Small and Di Feliceantonio [5] found that the method of food preparation and processing, apart from contributing to energy density or palatability, may also affect physiology, promote overeating and thus result in metabolic disorders. Other research also showed that ultra-processed foods may lead to overeating and facilitate the development of obesity or type 2 diabetes due to their high energy value and appetitive properties [6,7]. 

It should be noted that an increasingly industrialized food system is highly effective in terms of large-scale production and low prices, and it is difficult to replace it as it offers attractive prices and convenience, a long shelf-life, microbiological safety, and thus proper nutritional value [8]. Therefore, various institutions and non-governmental organizations issue nutritional recommendations in order to protect consumers from the development of non-communicable diseases and to reduce their symptoms [9]. Ultra-processed food consumption seems to be inevitable due to many factors including convenience, low prices and efficient marketing, but also the possibility of virtually unlimited enrichment with biologically active ingredients. Thus, it creates the illusion that the time saved contributes to consumers well-being, nevertheless, the price to be paid for the convenience can be high. Ultra-processed food is subjected to multidirectional processes and modifications prior to consumption and contains significant amounts of added sugar, salt, saturated fat and number of additives per product. Currently, there is no clear classification system or definition describing processing levels and categories reflecting real nutrient loading, which could increase applicability and efficiency in public health improvement [10].

According to Monteiro et al. [9,11], ultra-processed food has been defined as the result of a series of processing operations carried out in order to obtain a formulation mostly from cheap sources of energy, nutrients and selected additives, thus containing minimal whole foods. The NOVA food classification system has been among those most often applied in scientific research, however, it may not sufficiently identify foods with high nutrient quality commonly consumed by children in the U.S. [10]. It categorizes foods and beverages into four groups: unprocessed or minimally processed foods, processed culinary ingredients, processed foods and ultra-processed foods. NOVA ultra-processed food classification includes food substances hardly used in home cooking, but also food additives intended to improve the palatability and attractiveness of the final product.

Particularly interesting and unique results were presented by Rauber and her team [12]. They presented the impact of ultra-processed foods consumption on diet’s nutritional quality, known to affect the incidence of chronic non-communicable diseases. The authors focused on analyses of cross-sectional data from the U.K. National Diet and Nutrition Survey over the years 2008–2014. It was confirmed that frequent consumption of ultra-processed food causes an increase in free sugars, carbohydrates, total and saturated fats, as well as sodium supply in daily diet, thus increasing the risk of several diet-related diseases. Moreover, it should also be highlighted that a significant inverse linear relationship was found between the composition of ultra-processed foods and the protein, fiber and potassium content in the diet. Numerous recent studies have confirmed a similar strong association between the consumption of ultra-processed foods and dietary nutrient profiles affecting the welfare and health of the consumer. The negative effects of ultra-processed food consumption on overall diet quality, previously observed in Canada [1], the United States [13], and Brazil [14], have also been confirmed in the UK. Moreover, a high percentage of energy derived from a diet based on ultra-processed foods has been confirmed. Similar trends were not confirmed in countries dominated by traditional diets based on freshly prepared meals. An important finding of Rauber’s team’s research [12] is that a significant percentage of UK population did not meet the WHO recommendations for the prevention of non-communicable diseases in terms of dietary fiber, saturated fat, free sugars, potassium and sodium in the diet. On the other hand, it is suggested that the data presented in the publication may be subject to social bias. Moreover, the use of dietary diaries, considered as one of the most comprehensive methods of food consumption assessment, may limit the under-reporting of certain foods, unhealthy foods in particular, and therefore the overall consumption of certain nutrients. Nevertheless, the applied research methodology provides probable use in similar international contexts. 

In sum, average consumption in the UK is alarmingly high, reflecting the prevalence of ultra-processed products on the food market and their aggressive promotion. That situation results in excessive consumption, leading to obesity and other diet-related chronic non-communicable diseases. Therefore, broad-based action of governments to promote consumption of less processed foods with a healthier nutrient profile is becoming critical.

Those extensive studies were also followed by other research. Leite et al. [15] assessed the nutritional profile of foods and non-alcoholic beverages advertised on TV and indicated that the Brazilian population is highly exposed to unhealthy food marketing and inefficient enforcement of the existing legislation aimed to protect consumers health. Other research by Coyle et al. [16] demonstrated the ineffectiveness of an intervention which was based on supplying comparative nutrition data to a supermarket retailer in order to achieve improvements in the nutrition value of their private label products. This confirmed that there are other factors influencing the decision-making process, including final cost, flavor, and product sales impact. In turn, studies on ultra-processed foods’ consumption in Australia indicated that the nutrient intake is linked to non-communicable diseases and that the decrease in the dietary share of ultra-processed foods would substantially improve diet quality [17]. 

Hall et al. [18] investigated adults who consumed ultra-processed and unprocessed foods for 14 days each, in random order. The foods were matched in terms of nutritional value, including sugar, fat, fiber, macronutrients and calorie content. It was found that an ultra-processed ad libitum diet resulted in increased energy intake and weight gain. Based on those results, it is recommended to limit the consumption of ultra-processed foods in order to reduce the risk of obesity. The association between the consumption of ultra-processed foods and adiposity have also been examined by Rauber et al. [19]. The report compiled by the research team showed that a higher consumption of ultra-processed food is associated with greater adiposity in the UK adult population, and therefore the policy makers should promote unprocessed or minimally processed foods among the consumers. Particular attention should also be paid to the recent research conducted in a population of Montreal in Canada, which suggest the link between an increased risk of prostate cancer and a higher intake of processed foods [20].

It is impossible to overestimate the fact that governments and public health experts have noticed the necessity of taking action in order to play a greater role in preventive nutrition and health promotion. Such action includes recommendations to limit the amount of salt added to processed foods, remove trans-fatty acids from food, and to order schools to stop supplying unhealthy food to students [21].

In conclusion, recent years have presented observations regarding considerable interest in diet composition in relation to its beneficial nutritional value and health implications. Numerous studies suggest that increasing the consumption of freshly prepared, unprocessed or minimally processed foods with a simultaneous reduction in the share of highly processed foods in the diet would definitely have beneficial effects. The published research [12] presents an in-depth analysis of the potential impact of the above-mentioned measures, notably in relation to improving the nutritional quality of the diet and contributing to the prevention of chronic non-communicable diseases associated with diet. Such knowledge provides a high probability of its direct application in other regions of the world. The most important achievement of those studies is a universal guideline, indicating the need for radical strategies to reduce the consumption of ultra-processed foods by the entire population aimed at the prevention of diet-related non-communicable diseases.
